# What Is Known about the Immune Response Induced by *Plasmodium vivax* Malaria Vaccine Candidates?

**DOI:** 10.3389/fimmu.2017.00126

**Published:** 2017-02-13

**Authors:** Carolina López, Yoelis Yepes-Pérez, Natalia Hincapié-Escobar, Diana Díaz-Arévalo, Manuel A. Patarroyo

**Affiliations:** ^1^Molecular Biology and Immunology Department, Fundación Instituto de Immunología de Colombia (FIDIC), Bogotá, Colombia; ^2^PhD Programme in Biomedical and Biological Sciences, Universidad del Rosario, Bogotá, Colombia; ^3^MSc Programme in Microbiology, Universidad Nacional de Colombia, Bogotá, Colombia; ^4^Universidad de Ciencias Aplicadas y Ambientales (UDCA), Bogotá, Colombia; ^5^Basic Sciences Department, School of Medicine and Health Sciences, Universidad del Rosario, Bogotá, Colombia

**Keywords:** malaria, *Plasmodium vivax*, immune response, antigenicity, immunogenicity

## Abstract

Malaria caused by *Plasmodium vivax* continues being one of the most important infectious diseases around the world; *P. vivax* is the second most prevalent species and has the greatest geographic distribution. Developing an effective antimalarial vaccine is considered a relevant control strategy in the search for means of preventing the disease. Studying parasite-expressed proteins, which are essential in host cell invasion, has led to identifying the regions recognized by individuals who are naturally exposed to infection. Furthermore, immunogenicity studies have revealed that such regions can trigger a robust immune response that can inhibit sporozoite (hepatic stage) or merozoite (erythrocyte stage) invasion of a host cell and induce protection. This review provides a synthesis of the most important studies to date concerning the antigenicity and immunogenicity of both synthetic peptide and recombinant protein candidates for a vaccine against malaria produced by *P. vivax*.

## Introduction

Malaria is one of the most important vector-transmitted diseases, affecting a large part of the world’s population. Around 214 million new cases appeared in 2015, and 438,000 people died from the disease. This disease is caused by parasites from the phylum Apicomplexa, genus *Plasmodium*, and is transmitted by the bite of a female mosquito from the genus *Anopheles* infected by the parasite (1).

Five species cause malaria in humans: *P. falciparum, P. vivax, P. malarie, P. ovale*, and *P. knowlesi*. Acute febrile disease symptoms appear 10–15 days after the bite of an infected mosquito. Initial symptoms include fever, headache, shivering, and vomiting; if not treated early on, and depending on the species responsible for the disease, severe anemia, metabolic acidosis, or cerebral malaria may be produced, and even lead to death (1).

Studying the proteins involved in *P. vivax* invasion has not been easy, mainly due to technical restrictions such as a lack of continuous culture *in vitro*, meaning that studying the parasite’s biology has been limited, as well as the identification of new antigens and their evaluation *in vitro* (2, 3).

Infection by more than one *Plasmodium* species is usually omitted in routine diagnosis by microscopy (4, 5), leading to an overestimation of the amount of cases caused by coinfection in endemic areas and thus to treatment failure (6). Drug resistance since the first report in 1989 (7) has been increasing worldwide throughout Southeast Asia [Indonesia, China, Thailand, Papua New Guinea (PNG)], South America (the Brazilian and Peruvian Amazon region, Colombia), Africa (Madagascar, Ethiopia), Pakistan, and Turkey (8, 9).

Such resistance appears to be related to mutations regarding multidrug resistance 1 (mdr1) gene and variation in the gene’s number of copies, presumably due to selective pressure by first-line chloroquine treatment (10, 11).

Even though malaria caused by *P. vivax* has been considered benign (unlike that caused by *P. falciparum*), severe *P. vivax* malaria has emerged during the last few years with some cases leading to death (12–17). In spite of *P. vivax* malaria having a greater global distribution, it is still considered a neglected infection, thereby leading to socioeconomic impact factors being understated in endemic regions, causing more than US$2 billion per year costs worldwide (18). The forgoing means that investment and efforts must be focused on developing a vaccine against *P. vivax* malaria.

**Figure 1:**
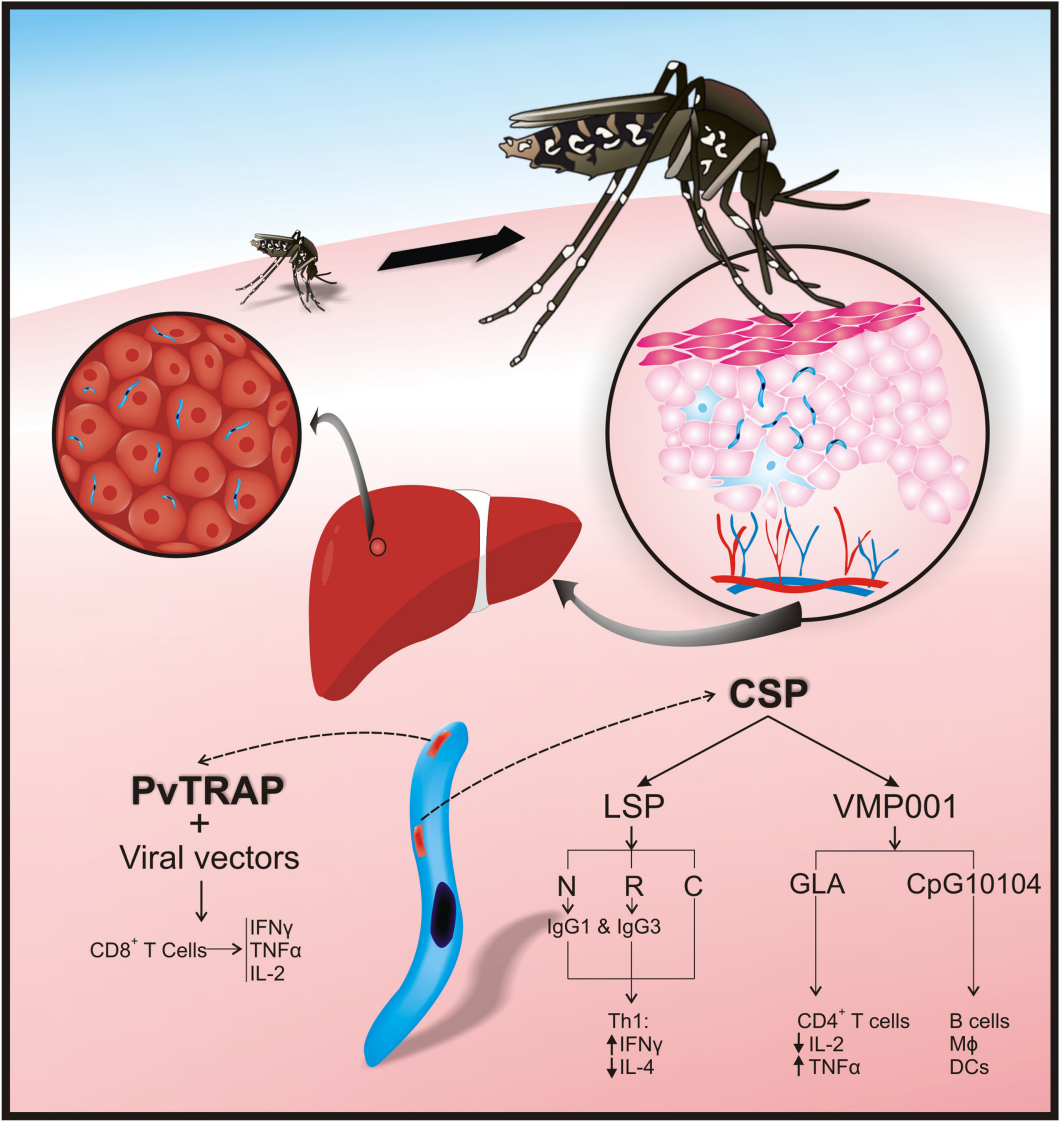
***Plasmodium vivax* preerythrocyte stage protein immunogenicity**. After sporozoites have been inoculated into the skin by *Anopheles* mosquitoes, they travel to the liver *via* the bloodstream and enter hepatocytes thereby initiating the preerythrocyte stage. *P. vivax* circumsporozoite protein (CSP) and thrombospondin-related adhesive protein (*Pv*TRAP) are involved in hepatocyte recognition and binding in a mammalian host. In CSP, the N-terminal (NT) and repeat region facilitate parasite binding to hepatocytes. Adaptive immune responses against *Pv*CSP and *Pv*TRAP control invasion of hepatocytes by cytokines [CD4+ T-helper 1 (Th1) and CD4+ T-helper 2 (Th2) cells], cytophilic antibodies, and CD8+ T-cells. Interferon gamma (IFN-γ) increases and interleukin (IL)-4 decreases after vaccination with CSP-long synthetic peptides [CSP-LSP-N terminal; CSP-LSP-R (repeat region), and CSP-LSP-N terminal]. Cytophilic antibodies (IgG1 and IgG3) are produced after vaccination with CSP-LSP-N; CSP-LSP-R. Immunization with *Pv*CSP recombinant vaccine (VMP 001) combined with CpG10104 has induced protection and activation of B-cells, macrophages (MΦ), and dendritic cells (DCs). When this recombinant vaccine is formulated with glucopyranosyl lipid A (GLA), there is activation of CD4+ T-cells, production of tumor necrosis factor-alpha (TNF-α), and reduction of IL-2. Immunization with *Pv*TRAP, expressed in viral vectors, induces activation of CD8 T-cells and production of IFN-γ, TNF-α, and IL-2.

**Figure 2:**
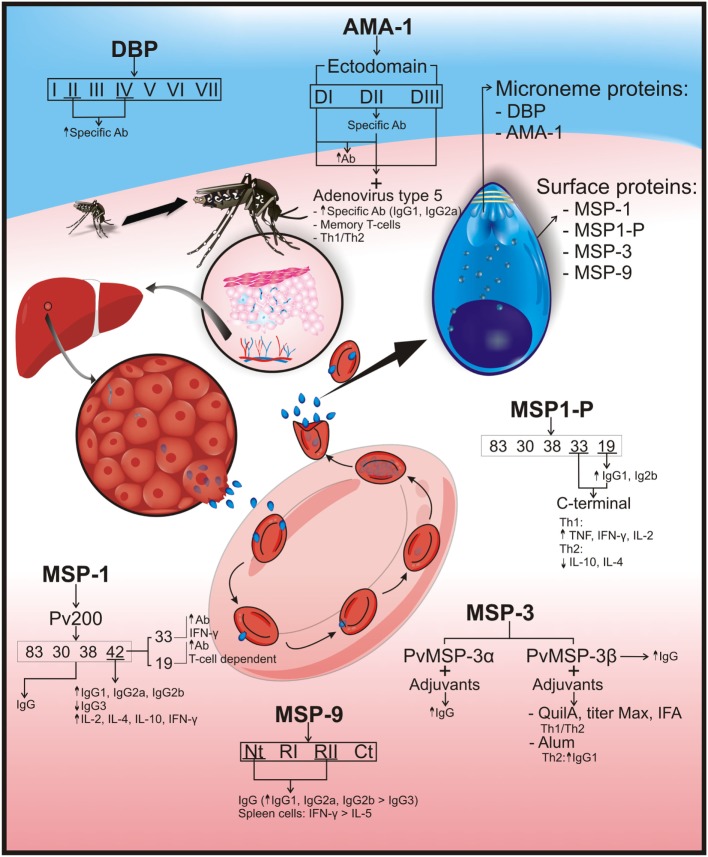
***Plasmodium vivax* erythrocyte stage protein immunogenicity**. *P. vivax* parasites are differentiated into tissue schizonts in hepatic cells, which, after thousands of replications, are released into the bloodstream as merozoites (Mrz). These Mrz predominantly invade reticulocytes, and their infection cycle is repeated every 48 h. Several surface and microneme merozoite proteins have been identified as vaccine candidates. Surface proteins would include merozoite surface protein-1 (MSP-1/*Pv*200), which is an abundant ligand on merozoite surface and is essential for reticulocyte invasion. MSP-1 was cleaved into 83, 30, 38, 42 (33 and 19) kDa fragments; immunization with the complete protein induced IgG production. MSP-1–42 fragment increased IgG1, IgG2a, and IgG2b production but not that of IgG3, as well as interleukin (IL)-2, IL-4, IL-10, and interferon gamma (IFN-γ) production in vaccinated mice. Immunization with 19-kDa fragments produced high antibody titers that were T-cell dependent. Higher antibody and IFN-γ production was observed after vaccination with the 33-kDa fragment. Another surface protein is merozoite surface protein-1 paralog (MSP1-P), which was also cleaved into 83, 30, 38, 42, 33, and 19 kDa; the last two fragments (C-terminal region) induced a Th1 cytokine response profile, having high tumor necrosis factor (TNF), IFN-γ, and IL-2, but low IL-10 and IL-4 cytokines (Th2 profile). High IgG1 and IgG2b titers were observed in vaccinated animals with 19-kDa fragment. Merozoite surface protein-3 (MSP-3): *Pv*MSP3-α block II is highly immunogenic and induces IgG production. The *Pv*MSP-3β region with Quil A, Titer Max, or IFA adjuvants has produced a balanced Th1/Th2 response and IgG but became directed toward a Th2 response when formulated with Alum. Merozoite surface protein-9 (MSP-9) immune response against NT and repeat region II was mainly IgG, having greater IgG1, IgG2a, and IgG2b titers than IgG3 isotype production. These two regions also induced higher IFN-γ than IL-5 production in spleen cells. The following are microneme proteins: the Duffy binding protein (DBP) is a surface receptor for invading human reticulocytes and is divided into seven regions where regions II (main ligand domain) and IV induce specific antibody production. Apical membrane antigen-1 (AMA-1) is essential during cell host invasion, and its ectodomain defines three subdomains (DI, DII, and DIII). Immunization with AMA-1 induced high IgG antibody titers. Vaccinating mice with human adenovirus type 5 and rAMA-1 have produced long-lived specific antibodies (IgG1 and IgG2a), memory T-cell, and Th1/Th2 balance immune responses. An arrow pointing upwards (↑) indicates an increase in antibody titers or cytokine production; an arrow pointing downwards (↓) shows reduced cytokine or antibody production.

Antigenicity studies arise from evaluating the immune response induced in individuals naturally exposed to the infection. On the other hand, immunogenicity assays evaluate *in vitro* or *in vivo* the immune response induced when vaccine candidates are used for immunization (Figures 1 and 2).

The present review summarizes classical studies that have been carried out to date concerning the antigenicity and immunogenicity of the most important proteins considered candidates for a vaccine against *P. vivax* malaria. Although the use of a single-stage protein is not enough to provide a successful sterile vaccine, it has represented an important advance in identifying hundreds of malarial antigens that can be combined to develop a multistage, multi-epitope sterile vaccine.

## Malaria: Infection by *P. vivax*

Around 90% of the clinical cases presented are the result of infection by two of the most relevant species: *P. falciparum* or *P. vivax*. *P. vivax* malaria is the second most important around the world and is the most prevalent on the Asian and American continents. Such infection is characterized by relapses several years after the first infection, since a latent form called hypnozoite occurs during hepatic phase. This stage is difficult to diagnose, allowing the parasite to survive in the host for longer (1, 19, 20).

Infection begins with the vector inoculating sporozoites (Spz) into the host’s skin; these Spz are motile and travel through the blood stream, later being carried to the liver. Sinnis et al. have named a “skin stage” of infection because they have proposed that this interaction between Spz and cells at the injection site means that Spz may remain in the injection site for 2–3 h, maybe in hair follicles, giving rise to infective merozoites (Mrz) (21, 22). Regarding *P. berghei* expressing GFP (a rodent parasite), it has been observed that Spz have a random gliding-movement. Moreover, Spz glide into the skin, interacting with blood vessel walls. Lymphatic vessels also become invaded to drain lymph nodes near the injection site where some Spz can partially develop into exoerythrocytic stages (23–25).

Sporozoites migrate from the skin to liver cells (these becoming infected first) and then cross/traverse endothelial cells and use cell traversal machinery to pass through the endothelium, thereby beginning the hepatic stage that might go unnoticed clinically (26, 27). Some parasites remain as hypnozoites during this stage, and others go into the blood stream giving rise to the erythrocyte stage where the disease’s clinical manifestations are presented.

The severity of the disease during the erythrocyte stage depends on various factors, such as the location of parasitized red blood cells (RBC) in the target organs, the local and systemic action of the parasite’s bioactive products, pro-inflammatory cytokine production, as well as innate and adaptive immune system cytokine and chemokine regulators, and the activation, recruiting, and infiltration of inflammatory cells (28).

After invading the hepatocytes, each Spz replicates within the parasitophorous vacuole by a family of parasite proteins having an NT export motif called pexel/VTS (*Plasmodium* export element/vacuolar translocation signal) (29–31). The circumsporozoite protein (CSP) enters the hepatocyte’s cytoplasm using the pexel/VTS motif and a nuclear localization signal to go into the nucleus. CSP in the nucleus induces the expression of the host’s genes, where the NFkβ transcription factor controls the expression of genes involved in inflammation (32), controlling biological functions such as metabolic transport, the cell cycle, the immune response, and apoptosis, thereby creating a favorable setting for parasite growth (31).

The *Plasmodium* erythrocyte stage begins when an infected hepatocyte ruptures and releases close to 30,000 Mrz into the blood stream, undertaking an initial journey as merosomes to the lungs and then becoming disseminated in the circulation. Each Mrz infects an immature RBC (reticulocyte), which generates 16 new Mrz 48 h later (33).

## Immune Response Regarding Malaria

Innate and adaptive immune system molecules are involved in disease pathogenesis and control. Clinical immunity to malaria can be acquired during three phases: immunity to the disease, immunity to symptomatic infection, and partial immunity to parasitemia (28). The premunition (absence of fever with infection and lower densities of parasitemia) is present in places where malaria is endemic and in people that had suffered of several infections through the years (8–15 years), thus acquiring natural immune responses that lower the risk of clinical disease (34). The term was coined early in the 1900s during epidemiological studies with patients from endemic areas that can control the parasitemia and develop a subclinical infection (35). It is characterized by a slow acquisition rate, present just in holo- or hyper-endemic areas, rapidly lost, strain dependent, IgG dependent, and directed toward blood-stage parasites; although the immune response induced is strong, it is not a sterilizing immunity. The protection mechanism has not been completely described, but there is evidence that cytophilic antibodies and memory cells produced after repeated infections with *Plasmodium* variants are responsible for this kind of protection (34, 36–39).

An innate immune response is triggered during *Plasmodium* infection as first line of defense, followed by an adaptive immune response, which includes T-cells, B-cells, and antibodies. A mosquito inoculates Spz into a host’s skin when biting; these can remain in the skin for up to 6 h after inoculation (40). Such retention affects the place for antigen presentation and the location and type of response so induced.

Dendritic cells (DCs) present antigens, depending on the anatomic environment and the resulting immune response. These cells, through pattern recognition receptors, recognize the pathogen-associated molecular patterns (PAMPs) exhibited by the parasite. The mechanism of action regarding such recognition triggers intracellular signals enabling DC maturation (41). Three PAMPS have been described in *P. falciparum*: hemozoin, immunostimulatory nucleic acid motifs, and glycosylphosphatidylinositol anchors [glycophosphatidylinositol (GPI) anchors] (42).

The parasite’s main source of protein is RBC hemoglobin. Hemoglobin hydrolysis releases lipophilic prosthetic group—heme—which is extremely toxic for the parasite. Heme detoxification is thus necessary and is achieved by converting heme into an insoluble crystalline material called hemozoin (Hz) (43). Regarding *P. falciparum* infection, Hz binds DNA inside host cell phagolysosomes and cytosol, and toll-like receptor (TLR)9 is activated by nucleic acids, NLRP3, AIM2, and other cytosolic sensors (42, 44–46).

In terms of AT content in the genome, *P. falciparum* has the highest AT content (82%) and *P. vivax* the lowest AT content (56%). On the other hand, *in silico* analysis has shown that *P. falciparum* contains ~300 CpG and ~6,000 AT-rich motifs and *P. vivax* ~2,000 CpG and ~5,500 AT-rich motifs. The release of CpG *Plasmodium* DNA into phagolysosomes produces an innate immune response activating TLR9 (42, 44, 47).

Glycophosphatidylinositol anchors connect surface proteins with the protozoan plasma membrane; they are essential toxins for parasite viability (48). They turn on the innate response because they induce cytokine synthesis and are recognized by TLRs such as TLR1–TLR2 or TLR2–TLR6 (depending on GPI anchor activation containing three or two fatty acid chains, respectively) and TLR4 (49–51).

Some functions described for DCs have been T- and B-lymphocyte activation, immune tolerance, natural killer (NK) cell activation, and macrophage activation (52). For example, a third of Spz are drained to regional lymph nodes where they become internalized by skin-derived DCs (CD103+) and presented to CD8+ T-cells (26).

Some studies have shown that infected RBC bind to DCs, inhibit their maturation, and cannot stimulate a T-lymphocyte response in acute *P. falciparum* infection. Other studies have shown that inhibition depends on contacting a larger amount of infected RBC per DC (53, 54).

Antigens begin to be presented on hepatocyte surface during the hepatic stage in context of the major histocompatibility complex (MHC) class I molecules expressed on all nucleated cells to become recognized by CD8+ T-cells (55).

Immune response during the erythrocyte stage is mainly mediated by antibodies while a cellular response predominates during the hepatic stage (56). CD4+ T-, B-, and NK cells also play an important role in the immune response induced by the parasite during the erythrocyte stage since immunity depends on memory B-cell production and lifespan, following infection (57).

During *P. vivax* infection, some individuals can acquire immunity naturally; such immunity consists of a cytokine production-mediated cellular immune response, cytokine receptors, and proteolytic enzymes forming part of the host response to infection, as well as IgG antibodies. Patients having moderate parasitemia in endemic regions of Colombia have high IFN-γ and TNF-α levels, a pro-inflammatory cytokine profile correlated with the response found in an unstable transmission region. The balance in interleukin (IL)-10/TNF-α rate could prevent increased parasitemia and host pathology (58).

A study by Hemmer et al. evaluated the production of lactate dehydrogenase (as hemolysis parameter), TNF-α (which produces a response to parasite products and has antiparasitic activity), thrombin–antithrombin III (pro-coagulant activity parameter), and human neutrophil elastase (its secretion becoming increased by parasite products, having antiparasitic activity) in a population infected by *P. falciparum* and *P. vivax* or *P. ovale* (59).

The parasitemia/response rate of each parameter was greater in patients suffering *P. vivax* or *P. ovale* malaria than *P. falciparum*; regarding parasitemia, TNF-α response was stronger in *P. vivax* or *P. ovale* infection than *P. falciparum*. The increase of these factors in *P. vivax* infection helped to control parasitemia, while they led to complications in *P. falciparum* infection such as host response-mediated severe malaria (59). Studies have shown that *P. falciparum* parasitemia levels decrease when coinfected with either *P. vivax* or other *Plasmodium* species, compared to single infection (60), thereby attributing a possible attenuating role to other *P. falciparum* species (60–62).

Another study that compared the immune response of patients suffering complicated and uncomplicated malaria caused by *P. vivax* reported a higher IFN-γ/IL-10 rate in patients having complicated disease, as well as higher TNF-α level. They concluded that the severity of disease caused by *P. vivax* was correlated with pro-inflammatory immune response activation and cytokine imbalance (63).

Interleukin-10 acts as immunoregulator by controlling the effects of other cytokines produced by CD4+ Th1 and CD8+ T-cells in *Plasmodium* infection. The overproduction of cytokines such as IFN-γ by these cells not only helps to increase phagocytosis for eliminating the parasite but also produces immunopathological effects associated with the disease. However, studies involving another group of patients suffering *P. vivax* malaria found high levels of IFN-γ and IL-10 in patients with previous episodes of malaria. Polymorphism studies regarding the IL-10 gene promoter in populations from endemic regions have shown that polymorphisms neither influence the production of this cytokine nor its regulatory function regarding the immune response (64, 65).

Goncalves et al. evaluated the cytokine pattern in uncomplicated symptomatic *P. vivax* and *P. falciparum* infection in a low malaria transmission region in Brazil to test the hypothesis that *P. vivax* infection causes a greater pro-inflammatory cytokine response than infection by other *Plasmodium* species. They found a greater anti-inflammatory cytokine response than in *P. falciparum* infection, but similar pro-inflammatory cytokine response. The response of anti-inflammatory cytokines such as IL-10 and IL-10/TNF-α, IL-10/IFN-γ and IL-10/IL-6 ratios in clinical malaria caused by *P. vivax* was short-lived and positively correlated with parasitemia rather than with the symptoms. This means that there must be a balance between inflammatory cytokine and regulator responses (66).

Network analysis was one of the approaches adopted by Mendoca et al. for understanding the interaction between different blood biomarkers for inflammation, tissue damage, and oxidative stress and the immunopathogenesis of malaria. They concluded that when studying uninfected individuals from endemic regions, the network of interactions showed high density between these biomarkers, limiting the symptoms but not the infection. IL-10 and IL-4 have connections with chemokine (C–X–C motif) ligand 9 (CXCL9) in uninfected people and with chemokine (C–C motif) ligand 2 and IFN-γ in people having asymptomatic *P. vivax* infection (67, 68).

Such interactions revealed a protective role for IL-4 and IL-10 cytokines due to their modulating effect on these pro-inflammatory cytokine and chemokine. The network of biomarkers in patients suffering mild malaria consisted of IFN-γ, tumor necrosis factor (TNF), and chemokine (C–C motif) ligand 5, while the interaction occurred between CXCL9 and IL-12 in symptomatic patients. CXCL9 was associated with regulatory cytokines thereby suggesting their role in resistance to infection, as well as being associated at the beginning with symptoms when linked to IL-12. Patients who had a fatal outcome regarding the disease had an interaction between TNF, IFN-γ, and IL-10, the latter modulating the Th1 response, negatively regulating TNF and IFN-γ, due to an IL-12p70 suppression that could lead to death (67, 68).

Cytokine profile variations have been observed in malaria–dengue coinfection. TNF levels have increased in patients with coinfection regarding single infection thereby highlighting the role of IL-6, INF-γ, and IL-7 (68). Regarding the humoral response, antibodies play an import role in protection against malaria, and this has been demonstrated in different studies. Cohen et al. showed that passive transfer of antibodies from malaria-immune individuals to naïve young children suffering severe clinical malaria reduced the parasite density and clinical symptoms related to this disease (69). This experiment was confirmed by further studies where adult patients controlled the clinical symptoms and parasitemia after receiving intravenously injections of sera from people living in malaria-endemic areas (70, 71).

Immunoglobulins can protect or arrest disease progression in different ways; neutralizing anti-Spz antibodies can block Spz from invading hepatocytes (72–74). Mrz can be opsonized in the erythrocyte stage by specific antibodies that activate cell-mediated death or prevent the invasion of RBC and block the proteins responsible for binding to molecules on cell surface.

Studies in malaria-endemic areas have suggested that high IgG3 and IgG1 cytophilic antibody titers are associated with protection (75). *In vitro* studies have shown that monocytes can kill asynchronic malaria parasites in the presence of cytophilic IgG3 and IgG1 antibodies (76). These antibodies facilitate phagocytosis and kill *Plasmodium* parasites since cross-linked FcγR–Fc induce a respiratory burst. Antibody responses against *P. vivax* CSP-1 (77), *Pv*MSP-1 (78–81), *Pv*RBP1 (82, 83), *Pv*AMA-1 (84), and *Pv*MSP-3α (85, 86) have been characterized by IgG1 and IgG3 predominance, which are associated with malaria exposure and malaria protection; Tables 1 and 2 summaries the results obtained in each study.

**Table 1:** ***Pv*CSP peptide antigenicity**.

Antigen	Sequence	Country	*N*	Prevalence of individuals having anti-antigen reactivity (IgG)	Reference
N	SSILLVDLFPTHCGHNVDLSKAINLNGVNFNNVDASSLGAAHVGQSASRGRGLGENPDDEEGDAKKKKDGKKAEPKNPREN KLKQPG	Colombia	80	35%	(87)
R	GDRADGQPA	Colombia	80	61%	(87)
C	YLDKVRATVGTEWTPCSVTCGVGVRVRRRVNAANKKPEDLTLNDLETDVCTMDKCAGIFNVVSNSLGLVILL	Colombia	80	39%	(87)
*Pv*CS-NRC	KAEPKNPREN KLKQPGDRAD	Colombia	42	58%	(88)
	GQPAGDRADGQPA-PEG-KAEPKNPREKLKQPGENGAGDQPGANGAGNQPG-PEG-NNEGANAPNEKSVKEYLDKVRATVGTEWTPCSVTCGVGVRVRRRVNAANKKPEDLTLNDLETDVCTMDKCA	Papua New Guinea (PNG)	32	69%	
*Pv*NR1R2	THCGHNVDLSKAINLNGVNFNNVDASS	Colombia	36	81%	(89)
	LGAAHVGQSASRGRGLGENPDDEEGDAKKKKDGKKAEPKNPRENKLKQPGANGAGNQPGANGAGNQPGANGAGNQPGGDRADGQPAGDRADGQPAGDRADGQPA	PNG	42	24%	
VMP 001	Recombinant *Pv*CSP with two copies of VK210 repetitive region (GDRA[A/D]GQPA) and one of VK247 (ANGAGNGPG)	Thailand	50	82%	(90)
r*Pv*CSP-c	Recombinant *Pv*CSP with four copies of the VK210 repetitive region (Belem strain) and three of VK247 (PNG strain)	Brazil	40	65%	(91)
		Thailand/Korea	114	73%	(92)

**Table 2:** **Erythrocyte phase protein antigenicity**.

Protein	Protein region	Country	*N*	Prevalence of individuals having anti-antigen reactivity (IgG)	Reference
Merozoite surface protein-1 (MSP-1)	N-terminal (NT)	Brazil (Pará)	37	51.4%	(78)
	C-terminal			64.1%	

MSP-1	C-terminal (*Pv*200_18_—18 kDa fragment)	Republic of Korea (Northern Province of Kyunggi)	421	88.1% (IgG)	(93)
				94.5% (IgM)	

MSP-1	*Pv*200L	Colombia (Buenaventura)	69	52.2%	(94)

MSP-1	C-terminal (*Pv*200_19_—19 kDa fragment)	Turkey (Province of Sanliurfa)	82	69.5% (IgM)	(95)
				53.6% (IgG)	
				7.3% (IgA)	

Merozoite surface protein-1 paralog	C-terminal (*Pv*MSP1P-19)	Republic of Korea (Province of Gyeonggi Gangwon)	30	73% (IgG3)	(81)
	C-terminal (*Pv*MSP1P-33)			43% (IgG1)	
				57% (IgG1)	

*Pv*MSP3-α	Full length	Brazil	276	77%	(86)
	C-terminal			54%	
	NT			39%	

*Pv*MSP3-α	Full length	Brazil (Rondonia State)	282	78%	(85)
	Repeat block I			64%	
	Repeat block II			53%	
	C-terminal			54%	
	NT			39%	

*Pv*MSP3-α	Repeat block I	Papua New Guinea (PNG)	264	36%	(96)
	Repeat block II			38%	
	C-terminal			65%	
	NT			38%	

*Pv*MSP3-α	FP-1 (aa 359–798)	Brazil (Amazon region)	220	68%	(97)

*Pv*MSP3-β	FP-1 (aa 35–375)	Brazil (Amazon region)	220	26%	(97)
	FP-2 (aa 385–654)			64.5%	
	FP-3 (aa 35–654)			66%	

*Pv*MSP-9	*Pv*MSP9-Nt	Brazil (Ribeirinha, Colina)	306	74%	(98)
	*Pv*MSP9-RI-RII				
	*Pv*MSP9-Ct				

*Pv*MSP-9	*Pv*MSP9-NT V_147_-K_159_; V_438_-D_449_; K_325_-I_339_; P_434_-I_448_; A_443_-K_456_	Brazil (Rondonia state)	142	61.2% (IFN-γ)	(99)
				49% (IL-4)	

*Pv*MSP-9	*Pv*MSP9-Nt	PNG	183	45.9%	(96)
	*Pv*MSP9-RI-RII			8.7%	

Duffy binding protein (DBP)	DBPII-IV	PNG (Mandang)	100	60%	(100)

DBP	DBPII-IV	Colombia (Buenaventura)	92	40%	(101)

Apical membrane antigen-1 (AMA-1)	*PV*66/AMA-1	Brazil (North)	221	85% (IgG)	(102)
				48.5% (IgM)	

AMA-1	DI	Brazil (Amazonas region)	100	13%	(103)
	DII			65%	
	DIII			12%	
	DI–DII			60%	
	DII–DIII			58%	
	Ectodomain			70%	

AMA-1	*Pv*AMA-1	Iran	84	81%	(104)

AMA-1	*Pv*AMA-1	Brazil	1,330	52.5%	(105)

AMA-1	*Pv*AMA-1	Brazil	83	73%	(106)

*Pv*RBP1	Full length	Brazil (Rondonia state)	294	66%	(82)
	*Pv*RBP1_431–748_			41%	
	*Pv*RBP1_733–1407_			47%	

*Pv*RBP1	*Pv*RMC-RBP1	Brazil (Rondonia state)	253	47%	(83)
	*Pv*RBP1_23–751_			60%	

*Pv*RBP1	Coiled-coil and C-terminal peptides	Republic of Korea	16	68%	(107)
	Repeat sequence peptides			62%	
*Pv*RBP2	NT and repeat sequence peptides			68–87%	
	Coiled-coil and C-terminal peptides			62–68%

*Pv*RBP1	*Pv*RBP1a-34	Republic of Korea	104	34%	(108)
	*Pv*RBP1b-32			39%	

### MHC Molecules and the Immune Response

Major histocompatibility complex proteins have high polymorphism in human beings; antigen-binding capability varies from one allele to another, increasing or reducing their affinity (55). The forgoing is essential for developing an effective vaccine inducing a protective immune response.

Major histocompatibility complex antigen-presenting molecules are divided into two large groups. Class I present intracellular antigens and can couple eight to nine amino acid-long peptides due to the smaller size of their grooves. Class II recognizes extracellular antigens and can display 13–18 amino acid-long peptides (109).

The genes encoding class II MHC proteins in humans are called human leukocyte antigen (HLA) and are in chromosome 6. They have alpha and beta subunits, an immunoglobulin domain, and a short transmembrane portion. They have genes from three classes of protein: HLA-DP, HLA-DQ, and HLA-DR; the most polymorphic locus is HLA-DR at expense of a highly polymorphic beta subunit, unlike the alpha subunit, which is monomorphic in humans (110).

The peptide’s binding site is formed by two almost parallel alpha helix regions above a beta sheet. The peptides are bound in the groove formed by the helices, with their terminal residues extended. The peptides adopt an extended poly-proline type II conformation exposing the peptide backbone to MHC conserved hydrogen-bonding residues covering the groove. Such conformation allows a peptide’s side-chains to bind to the groove of the MHC binding site, as well as allowing the side-chains to bind to MHC pockets in positions 1, 4, 6, and 9. The others bind to the T-lymphocyte receptor (TCR)-binding site (55, 111).

Major histocompatibility complex class II molecules, expressed constitutively on antigen-presenting cell surface (DCs, macrophages, and B-lymphocytes) recognize extracellular peptides processed by the endosome/lysosome pathway, which are edited by the HLA-DM molecule. MHC class II presents the antigen to the TCR of CD4+ T-lymphocyte (T-helper) (55).

The CSP is one of the most important proteins described to date in Spz. Previous studies involving individuals residing in *P. vivax* malaria-endemic regions in Brazil have shown low responses for antibodies directed against the repeat region. An association has been reported between the HLA-DR16* allele and antibody response to *P. vivax* VK247 variant CSP repeats, as well as an association between the HLA-DR7* allele and a lack of antibody response to VK210 variant CSP repeats (112).

A study involving an infected population in Brazil evaluated the relationship between HLA-DRB1* alleles and the antibody response to CSP, MSP-1, AMA-1, and Duffy binding protein (DBP) peptides. A significant association was found between high MSP-1 antibody levels (especially to the *Pv*200L fragment) and the HLA-DR3* allele; while no association was found between CSP, AMA-1, and DBP antibody production and HLA-DRB1* alleles (113).

During the erythrocyte phase, the merozoite surface protein (MSP) family is the responsible of the interaction between Mrz and reticulocytes. *Pv*MSP-1, *Pv*MSP-3, and *Pv*MSP-9 are potential vaccine candidates since they are exposed to the immune system and are recognized by antibodies from naturally infected individuals. A study by Lima-Junior et al. evaluated IgG antibody response to *P. vivax* MSP-1, MSP-3α, and MSP-9; a relationship between HLA-DRB1*04 individuals and high antibody response to *Pv*MSP3_CT_ and *Pv*MSP3_NT_ and HLA-DQB1*03 individuals, and response to *Pv*MSP3_CT_ was observed (86).

IgG response was positively associated with HLA-DRB1*04 and HLA-DQB1*03 individuals regarding *Pv*MSP-9 repeat regions and the NT region. Such response involving high antibody levels was associated with a possible selective pressure by *P. vivax* in the Amerindian population. Antibody responses for *Pv*MSP-9 were more correlated with the time spent living in a malaria-endemic area and not with a particular HLA-DRB1* allele (86).

Ferreira et al. constructed and expressed a synthetic gene encoding promiscuous T-helper epitopes bound to the *Pv*RBP1_435–777_ sequence. Although it has been observed in clinical assays that candidates for a vaccine against *P. vivax* are poorly immunogenic, they predicted that this chimerical protein (called *Pv*RMC-RBP1) would be recognized by multiple HLA alleles. Epidemiological and serological studies proved the preservation of B-cell conformational epitopes in the chimeric protein. However, no association was found between HLA-DRB1* and HLA-DQB1* alleles and IgG antibody responses to the chimeric or native proteins. This seemed to be because *Pv*RBP1 had multiple promiscuous T-cell epitopes, which did not induce specific genetic restriction (83).

### Non-HLA Host Polymorphism

Miller et al. proved (for the first time) the hypothesis that the Duffy negative erythrocytes are resistant to *P. vivax* infection in Africans (114). The Duffy antigen or Duffy antigen receptor for chemokines (DARC) is expressed on RBC surface (115). Its encoding genetic locus having three alleles [FY*A (Fy^a^) and FY*B (Fy^b^) with SNP of differences and FY*O] has a negative serological phenotype Fy(a−b−) (116, 117). The absence of DARC expression in RBC is due to a point mutation (T46C) in the GATA box of this gene’s promoter (118, 119).

Duffy negative patients infected with *P. vivax* have been found during the last few years (56, 120). Mendes et al. reported that Duffy negative individuals from Africa’s West Coast were infected with different strains of *P. vivax*, and they concluded that the parasite evolved quickly and used other receptors different to Duffy to invade RBC (121).

The balance between innate and adaptive immune response is important in the development of immunopathology and clinical severity in several infectious diseases. Sohail et al. have investigated polymorphisms in the TNF-α gene promoter region and its association with vivax infection in an Indian population. They found that TNF-308A and TNF-1031C were associated with vivax infection (very low frequency) in the study population (122). Other research has shown that IL1B, IL4R, IL12RB1, and TNF genes were associated with susceptibility to *P. vivax* malaria in a population from Brazil’s Pará state, reporting −5,839C>T SNP promoter association with *P. vivax* malaria susceptibility (123).

Da Silva et al. have shown an Amazonian population’s many host polymorphisms association with susceptibility or resistance to malaria infection. SNPs in the IL-10, CTL4 and TLR4 genes have been significantly associated with lower risk of clinical malaria, while a SNP in the IRF1 gene has displayed an enhanced risk. An intronic SNP in LTA was associated with protection, and one SNP on the TNF promoter was associated with susceptibility to clinical malaria (124).

Another study found no differences regarding IL6-176G>C polymorphism distribution in participants making up the different clinical groups of vivax malaria in a Brazilian population. No association was found between TNF-308G and clinical manifestations of malaria and no haplotype having DDX39B (22 C>G and 348 C>T) and TNF-308G> was identified or polymorphisms increasing the risk of clinical vivax malaria. Moreover, study participants having the genotype combination described here associated with resistance against manifestations of *P. vivax* infection (CG/CC/GG/GG) also had lower levels of pro-inflammatory TNF and IL-6, suggesting that DDX39B confers protection against malaria pathogenesis by reducing inflammatory response (125).

## *P. vivax* Preerythrocyte Phase Protein Antigenicity and Immunogenicity

### *P. vivax* CSP

One of the predominant surface proteins in Spz is the CSP; it is expressed during the preerythrocyte phase and plays a fundamental role during hepatocyte invasion (126). This protein is a candidate for a vaccine against malaria in the preerythrocyte phase since various studies have shown that anti-CSP antibodies block hepatocyte invasion (88, 127, 128).

Circumsporozoite protein in the different *Plasmodium* species has a highly conserved structure; it consists of an NT extreme (N), a species-specific central repeat region (R) located between two conserved regions (region I and region II), and a GPI anchor in the C-terminal extreme (129). The repeat region contains an immunodominant B-cell epitope, which is associated with its immunogenic potential (130).

Furthermore RTS,S/AS01, the most advanced recombinant vaccine to date for preventing malaria caused by *P. falciparum*, is designed from *P. falciparum* circumsporozoite protein (*Pf*CSP) repeat region peptides and C-terminal region T- and B-epitopes, coexpressed with hepatitis B surface antigen. Phase III clinical studies have shown 33–50% efficacy 1-year post-immunization in 5- to 17-month-old infants (89, 92, 131). However, after a 7-year follow-up, the vaccine efficacy declined to 4.4%, with a 16.6% efficacy against all episodes of clinical malaria in the low-exposure cohort and to −2.4% in the high-exposure cohort (132). Furthermore, no vaccine efficacy was observed against severe malaria in children and young infants immunized with RTS,S/AS0 (133). Given that CSP has been widely studied in *P. falciparum*, and among different *Plasmodium* species, this protein is considered as a potential target for designing a vaccine against *P. vivax* (134–137).

Three allele variants of the *P. vivax* circumsporozoite protein (*Pv*CSP) have been described: VK210, VK247, and vivax-like CSP-P, which differ at repeat region sequence level (138, 139). VK210 has greater global distribution, being found in countries like Brazil (140), India (141), Thailand (142), and Peru (143), while VK247 is found in some regions of Colombia and Brazil (144), and vivax-like CSP-P in Brazil (140), Indonesia, Madagascar, and PNG (139).

Antigenicity studies in people exposed to the disease in different endemic regions have found variable prevalence in individuals responding to different *Pv*CSP fragments (87) (Table 1). Preclinical studies and phase I clinical assays (Table 3) have been carried out regarding *P. vivax* with long synthetic peptides (LSP) having more than 70 *Pv* amino acids from *Pv*CSP amino terminal (N), carboxyl terminal (C), and repeat (R) regions linked to tetanus toxoid peptide (87). Immunized non-human primates from the genus *Aotus* spp. produced specific antibody response recognizing both LSP and CSP since the first immunization (87). LSP has also induced a Th1-type immune response characterized by increased IFN-γ and reduced IL-4 production in T-lymphocytes stimulated *in vitro* (87, 137).

**Table 3:** ***Plasmodium vivax* vaccine clinical trials**.

Stage	Protein	Name	Type	Clinical trial	Reference
Preerythrocytic	*Pv*CSP	CSP-N, -R, -C	LSP	Phase Ib	(77, 145)
	*Pv*CSP	VMP001	Rec	Phase I, IIa	(146)
Transmission blocking	*Pvs*25	Sc*Pv*s25/ISA51	Rec	Phase I	(147)
	*Pvs*25	*Pv*s25H/Alhydrogel	Rec	Phase I	(148)
Erythrocytic	*Pv*DBP	ChAd63 *Pv*DBP	Viral vector	Phase Ia	www.clinicaltrials.gov NCT01816113

*LSP, long synthetic peptide; Rec, recombinant; CSP, circumsporozoite protein*.

High IFN-γ production *in vitro* and cytophilic antibodies (IgG1 and IgG3) capable of recognizing fragments from the *Pv*CSP N- and R-regions have been produced by LSP (as in experimental models) in phase I clinical assays. By contrast, the C-terminal region has not been immunogenic in humans (77).

Specific B- and T-cell epitopes must be included to stimulate an immune response thereby enabling recognition by class I and class II MHC molecules. Two modified LSP, *Pv*CS-NRC (137 aa) and *Pv*NR_1_R_2_ (131 aa) have induced a strong antigen-specific antibody response in immunized mice. They have inhibited Spz invasion of hepatoma cells (HepG2A-16) *in vitro* by 65 and 90% for both *Pv*CS-NRC and *Pv*NR_1_R_2_, respectively (88, 128). *Pv*CS-NRC has included conserved regions I and II and the repeat region sequence from VK210 and VK247 variants (88, 129). *Pv*NR_1_R_2_ has been improved by including B-epitopes, T-epitopes, and cytotoxic lymphocytes epitopes (128).

Immunogenic fragments of CSP have been evaluated in a recombinant vaccine (VMP 001) expressed in *Escherichia coli* encoding a *Pv*CSP chimera (149). VMP 001 has triggered a potent immune response in BALB/c mice following a third immunization. The antibodies so produced were capable of agglutinating live Spz, indicating a loss of Spz-infective capability (150). r*Pv*CSP-c, a recombinant protein similar to VMP 001 (91), has shown antibody-specific reactivity against variants VK210 and VK247 (151).

Formulations have been made with adjuvants or TLR agonists to maximize vaccine candidate fragments’ immune response. Regarding adjuvants, assays involving BALB/c mice and *Aotus* spp. monkeys have proved that formulation with Freund’s, Montanide ISA270, and Montanide ISA51 adjuvants, which have not led to significant differences concerning specific antibody production. However, clinical assays have revealed greater immunogenicity (having greater antibody titers and IFN-γ production) when Montanide ISA 51 adjuvant was used (145). Other adjuvant that has been tested is the inert nanoparticles, which is coated with the *P. berghei* CSP and induced CD8 T cell immunity without pro-inflammatory signals and also induced IFN-γ production levels determined to be required for sterile protection in the *P. berghei* challenge model (152).

*Pv*CSP has been formulated with TLR agonists helping to improve the immune response. *Aotus nancymaae* immunized with VMP 001 plus a TLR9 agonist (CpG 10104) have produced high antibody titers since the first dose, antibodies directed against the C-terminal region, and the VK210 variant repeat region predominating. It was seen that 66.7% of immunized primates became protected following experimental challenge (153), associated with the activation of B-cells, macrophages, and DCs by the CpG 10104 agonist (154).

Other formulations have been evaluated by using new agonists. When using VMP 001 for immunization with the TLR4 agonist (glucopyranosyl lipid A) it was found that this created a CD4+ cell response with high IL-2 production but low TNF levels (90). Fusing a polypeptide covering the *Pv*CSP immunodominant region coformulated with the FliC agonist (*Salmonella typhimurium* flagellin) produced a PAMPs-dependent immune response *via* TLR5 (155).

The results of *Pv*CSP vaccine phase I/II of *Pv*(VMP001/AS01B) have been published recently; it induced an antibody, cell-mediated immune response and delayed the latent period, but it did not induce sterile protection (146). Experience with *P. falciparum* vaccines has demonstrated that single-stage and single-target antigens cannot induce long-lived, sterile protection (133). The next generation *P. vivax* vaccine should include multiple targets, especially those needed for binding to host cells and those blocking transmission.

### Thrombospondin-Related Adhesive Protein (*Pv*TRAP)

The thrombospondin-related adhesive protein (TRAP) has also been evaluated as a potential preerythrocyte phase vaccine candidate. TRAP is a type I transmembrane protein, expressed in the micronemes and translocate to Spz surface during hepatocyte invasion. The ectodomain consists of an A domain, a thrombospondin type 1 repeat (TSR), and a repeat region, which is variable among species. The A and TSR regions are cell adhesion domains that interact with hepatocyte membrane receptors thereby enabling invasion (156–158).

This protein has been studied in *P. berghei* and *P. falciparum* as vaccine candidate; these studies showed a significant reduction in parasites during hepatic phase, mediated by CD8+ cytotoxic T-lymphocytes but involving low antibody production (159, 160) *P. falciparum* (161).

A *P. vivax* study with *Pv*TRAP LSP involved immunizing mice and *Aotus* spp., which were then experimentally challenged. A good antibody response against the peptide was produced in mice, but only 50% recognized Spz. Four immunizations were needed in *Aotus* for obtaining a significant antibody titer, but IFN-γ levels did not increase; four of the six monkeys became protected following experimental challenge (162).

Mice immunized with *Pv*TRAP expressed in viral vectors have induced a better immune response associated with high IFN-γ production and TNF-α by CD8+ T-lymphocytes and the production of high antibody titers specific against *Pv*TRAP. A marked increase in IL-2 production from CD8+ lymphocytes has been seen after inoculating Spz, indicating an active response in the liver (163).

One of the main problems in developing an antimalarial vaccine using *Pv*TRAP has been its high genetic polymorphism observed in different isolates from different regions around the world (164). An approach to preventing these problems would involve studying proteins’ conserved regions instead of using immune-dominant antigens, which are highly polymorphic.

## *P. vivax* Erythrocyte Phase Protein Antigenicity and Immunogenicity

### Merozoite Surface Protein-1 (MSP-1)

The MSP family has been the most studied candidate from the erythrocyte asexual phase when developing an effective vaccine against malaria. The MSP-1 belongs to this family, being one of the most studied and currently important for both *P. falciparum* and *P. vivax* (165).

The MSP-1 analog in *P. vivax* is encoded by the *Pv*200 gene (166), having a 200-kDa molecular weight (167). The proteolytic processing profile is thought to be similar than for *P. falciparum* MSP-1, leading to 4 fragments: 83, 30, 38, and 42 kDa; further cleavage of the last one (C-terminal region) produces 33 and 19 kDa polypeptides, which are released to the blood stream. A 19-kDa portion remained bound to the recently formed ring phase following reticulocyte invasion (79, 168).

A study in *Pv* exposed individuals found IgG responses to r*Pv*200L (like the *Pf*190L fragment) indicating that the protein has high antigenicity. Sera from immunized animals showed IgG-specific antibodies capable of recognizing this protein *Pv*. The authors highlighted the fact that the observed response had a protective tendency since *Aotus* spp. developed low parasitemia peaks following *P. vivax* challenge (94).

The 19-kDa C-terminal fragment has been one of the most studied from MSP-1 (*Pv*200). Kaslow and Kumar studied *Pv*200_19_ protein immunogenic capability in mice vaccinated with three doses. An increase in antibodies was observed in sera, which became increased with the second vaccination, this being attributed to a booster for helping epitopes in *Pv*200_19_. The response was T-cell dependent, suggesting that an immune response to a vaccine based on this protein could be boosted by natural infection (169).

Studies in an endemic area of Brazil by Soares et al. detected IgG antibodies against MSP-1 C-terminal and NT region. Response to the C-terminal region increased according to patients’ number of previous episodes of malaria, an increase of up to 80% being observed in patients who had suffered more than four episodes. Moreover, *in vitro* proliferation was observed in 47% of the individuals and IFN-γ production 54% of them. This study suggests that the C-terminal region contains two immunogenic epidermal growth factor (EGF)-like domains which induce T-cell and antibody responses against *P. vivax* during natural infection in humans (78). Later studies with the C-terminal region, specifically *Pv*MSP-1_19_, have shown that these two EGF-like domains function as a binding portion in *Pv*MSP-1 interaction with erythrocytes (170).

Later studies by Soares et al. found that antibody titers against *Pv*MSP-1_19_ became rapidly reduced (by up to 13-fold) in infected individuals and those who had received treatment against the disease. Antibody response against the NT region became reduced, even though such reduction was not significant. The decrease of antibodies directed against the C-terminal region could have contributed toward cases of reinfection in high-risk areas (171). Fernandez-Becerra et al. evaluated IgG subclasses in children, finding a predominant IgG1-type response against the C-terminal region and low IgG3 and IgG4 percentages. The predominant antibodies in adults were IgG3, a correlation between antibodies against the C-terminal region and age being observed (172).

High antibody titers against *Pv*200_19_ have been observed in infected soldiers in the republic of Korea *Pv*, mostly IgG and IgM to a lesser extent; these were maintained for a long period of time (from 4 to 6 months) following recovery from malaria (173). Another study showed that IgG antibody permanence was maintained for more than 5 months, while IgM-type remained negative 2–4 months after the onset of symptoms (93).

The major responses in Turkey (where *P. vivax* is the only *Plasmodium* species present in the area) were IgG, IgM, and IgA to a lesser extent. It is worth highlighting the fact that *Pv*MSP-1_19_ was highly antigenic in individuals who are naturally exposed to the infection and, since no other Plasmodia are infecting in that area, the response observed cannot be attributable to a crossed reactivity (95).

Rosa et al. characterized the MSP-1_19_ recombinant protein’s antigenic and immunogenic properties together with two T-helper epitopes (the universal pan allelic DR epitope and a new internal MSP-1 epitope from the 33-kDa C-terminal region). It was seen that T-helper epitopes did not modify protein recognition by human IgG. The complete recombinant protein was immunogenic in marmosets (*Callithrix jacchus jacchus*), but only when Freund’s adjuvant was used (174).

A study of immune response and protection was conducted in our institute, two groups of *Aotus* spp. (one splenectomized and the other not) were immunized with two recombinant polypeptides (r*Pv*MSP-1_14_ and r*Pv*MSP-1_20_) from the MSP-1 33-kDa C-terminal region containing high activity binding peptides (HABPs) to reticulocytes. Most immunized monkeys recognized the r*Pv*MSP-1_14_, r*Pv*MSP-1_20_, or the mix of HABPs by enzyme-linked immunosorbent assay (ELISA) and denatured *Pv*MSP-1 42 and 33-kDa fragments by Western blot. Although half the animals immunized with the r*Pv*MSP-1_14_ and r*Pv*MSP-1_20_ mixture were protected, some monkeys did not produce antibodies against the vaccine candidate. This suggested that protection was not only mediated by a humoral immune response (175). The next study involved vaccinating *Aotus* spp. monkeys with the two above mentioned recombinant polypeptides but in three doses. *Aotus* spp. produced antibodies capable of recognizing the native protein and managed to control parasitemia in four out of the five immunized monkeys. Interestingly, some animals produced high IFN-γ levels and controlled parasitemia but displayed low antibody titers; conversely, some other animals were protected having high antibody tires but low IFN-γ levels (176).

Other studies have found that anti-*Pv*MSP-1 antibodies (predominantly IgG), recognizing the NT region, are associated with a reduced risk of infection and clinical protection against the *Pv*MSP-1. Although these antibodies do not recognize the C-terminal region (79), there have been reports that the C-terminal region is immunogenic and capable of naturally stimulating antibody production in new infections (78, 93, 173).

The MSP-1 protein’s 42-kDa fragment has also been studied due to its potential as vaccine candidate; its immunogenicity was evaluated in mice. High IgG1, IgG2a, and IgG2b antibody levels were observed while IgG3-type response was low. A high proliferative response also found high IL-2, IL-4, IL-10, and IFN-γ levels being detected in culture supernatants (177). Greater prevalence of recognition of *Pv*MSP1_19_ than *Pv*MSP1_42_ was found when a naturally acquired humoral immune response was reported (178).

An MSP-1 paralog has been identified recently (*Pv*MSP1-P); its immune response was characterized using different protein fragments (83, 30, 38, 42, 33, and 19 kDa). The NT (83 kDa) fragment and two from the C-terminal region (33 and 19 kDa) were recognized by sera from infected patients living in endemic areas (179).

IgG1 and IgG3 (IgG2b in mice) were the predominant responses in patients from endemic regions, as in immunized mice. The C-terminal region induced a predominantly Th1 profile of cytokine response having high TNF, IFN-γ, and IL-2, but low levels of IL-10 and IL-4 cytokines (Th2 profile), showed greater lymphoproliferative response than the MSP1–19 fragment. The correlation between parasitemia and anti-MSP1P antibody level suggest that it does not contribute strongly to inhibiting parasite growth (81).

Due to its colocation with MSP1, it has been thought that it played a similar role in erythrocyte invasion; however, analyzing the sequences has suggested different roles for each protein. Cytoadherence assays demonstrated that MSP1-P could be an essential adhesion molecule regarding *P. vivax* invasion to erythrocytes, is immunogenic in humans, and is a potential vaccine candidate against *P. vivax* (179).

### Merozoite Surface Protein-3 (MSP-3)

The *P. vivax* merozoite surface protein-3 (*Pv*MSP3) is a member of the MSP family characterized by having a highly polymorphic alanine-rich central domain (180). It has a relatively conserved N- and C-terminal domain and two central blocks of seven repeats forming tertiary supercoiled helices in their structure (180–182). It is expressed in schizonts and is associated with Mrz surface during the erythrocyte phase (180).

Its homolog in *P. falciparum* has been studied as a vaccine candidate in preclinical (183) and phase I assays, protection was associated with reduced parasitemia by cytophilic antibodies inducing antibody-dependent cell-mediated inhibition of parasite growth mechanism (184). It has been shown to be highly immunogenic in *P. vivax*, having a high prevalence of antibodies directed against *Pv*MSP-3α block II (96).

A correlation has been described between time spent living in an endemic region and the number of previous episodes of malaria, involving an increase in IgG1 and IgG3 anti-*Pv*MSP-3α (85, 86, 96). A naturally acquired response has been found toward 15 antigenic determinants, mainly located in repeat regions (85). Other studies have reported the C-terminal region as being the most antigenic, having a significant increase in IgG in a population from Brazil (97) and PNG (96). However, it has been found the antibodies directed against block II are associated with protection against clinical episodes of *P. vivax* malaria, having greater than 500 parasites/μL parasitemia (96).

Interestingly, no response against *Pv*MSP-3α was produced in immunogenicity studies with C57BL/6 mice, while high antibody titers were produced with PvMSP-3β following the second and third immunization (97). Incorporating adjuvants (Quil A, TiterMax, or IFA) has maximized the response against *Pv*MSP-3α, indicating that another parasite’s molecules must act as adjuvant for *Pv*MSP-3α antigenic presentation during natural infection (96, 97). Regarding cellular response, *Pv*MSP-3β with Quil A, Titer Max, or IFA adjuvants have produced a balanced Th1/Th2 response, while the Alum adjuvant directed response toward Th2 with a significant murine IgG1 increase. Alum co-formulated with the TLR9 agonist (CpG ODN 1826) balanced the Th1/Th2 relationship, increasing Th1 response due to pro-inflammatory cytokine production (97).

### Merozoite Surface Protein-9 (MSP-9)

The *P. vivax* merozoite surface protein-9 (*Pv*MSP-9) is also a potential vaccine candidate. Some studies have shown that this protein is conserved among *Plasmodium* species infecting humans, rodents, and primates. Furthermore, antibodies produced against *Pv*MSP9 homologs in *P. cynomolgi* and *P. knowlesi* can inhibit Mrz invasion of erythrocytes (180).

The *P. vivax, P. knowlesi*, and *P. cynomolgi msp-9* genes encode a hydrophobic signal peptide and repeat motifs upstream of the stop codon and a C-terminal region having two species-specific blocks of repeat amino acids (*Pv*MSP9-RI and *Pv*MSP9-RII). Together with *P. falciparum* has four cysteine residues close to the NT giving the MSP-9 family’s structural and functional characteristics. This protein is expressed during schizogony and is organized on Mrz surface during schizont development and segmentation (180, 185).

The cellular and humoral immune response of BALB/c mice immunized with *Pv*MSP9-Nt, *Pv*MSP9-RII recombinants, and the mixture of both recombinants was evaluated for testing *Pv*MSP-9 immunogenicity. Antibody response in mice was determined by ELISA and was mainly IgG; there were greater titers for IgG1, IgG2a, and IgG2b isotypes than IgG3. Regarding cellular response, the amount of spleen cells secreting IFN-γ was higher than those secreting IL-5. Moreover, sera from patients living in an endemic region of Brazil recognized the two recombinant regions, demonstrating that both were immunogenic (186).

A study was carried out on a population, which was naturally exposed to *P. vivax* infection in Brazil, and the immune response against *Pv*MSP9-RIRII and *Pv*MSP-N terminal domains was evaluated. Of the 306 individuals in this study, 74% had IgG antibodies that recognized at least one of the recombinant proteins, thereby indicating that these proteins are antigenic during natural infection, especially *Pv*MSP9-RIRII. When the IgG subclasses were evaluated, IgG1 was predominant for *Pv*MSP9-RIRII and *Pv*MSP9-N terminus, and IgG2 was prevalent for *Pv*MSP9-RII. Furthermore, five synthetic peptides predicted to bind to HLA-DR alleles were chosen, and the overall cellular response frequency for at least one of the peptides was 58% for IFN-γ and 41% for IL-4. No association was found between IFN-γ production and IgG levels regarding recombinant proteins. Lima-Junior et al. thus concluded that *Pv*MSP9 C-terminal and NT domains are immune response targets for individuals living in *P. vivax* endemic regions. The reactivity index for IgG has been positively correlated with time spent living in an endemic area; conversely, IgG3 reactivity did not predominate regarding response to the recombinant proteins. It has been shown that *Pv*MSP9 NT region peptides induce memory T-cell response where IFN-γ and IL-4 cytokines produced in significant proportion by individuals from endemic regions has indicated the presence of T-cell epitopes (98).

Another study involving volunteers from an endemic region of the Amazon region showed that the response of cells producing IFN-γ was significantly greater than those regarding IL-4. The results obtained for 5 of the 11 peptides selected contained *Pv*MSP9 promiscuous T-cell epitopes. The core sequence (ASIDSMI) shared by three of the peptides was highly immunogenic; another peptide could have had two immunodominant epitopes, one in the overlapping core region and another in the C-terminal region, which produced a cellular response in 23 volunteers (99).

The specific response of IgG to *Pv*MSP9-N terminal has been associated with protection against symptomatic *P. vivax* infection in children aged less than 3 years old in a PNG endemic region. Such antibodies specific for *Pv*MSP9 were prevalent in children suffering frequent infections and have been associated with protection in children who have not had these infections. The authors concluded that two classes of antibodies are produced against the *Pv*MSP9 NT region, one produced by short-lived memory B-cells and the other by long-lived cells (96).

### Duffy Binding Protein (DBP)

*Plasmodium vivax* Mrz requires antigens from the Duffy blood group as surface receptor for invading human reticulocytes (114). *P. vivax* DBP adhesion to its receptor on erythrocytes [Duffy antigen receptor for chemokines (DARC)] is essential for the parasite to continue developing during the asexual phase in human blood (114, 187). *Pv*DBP is a 140-kDa protein, which is located in the micronemes; it has been divided into four important regions: a peptide signal sequence (region I), two cysteine-rich regions separated by a non-homologous hydrophilic region (region II, identified as the erythrocyte-binding domain, and region VI), and transmembrane domain (region VII) (188–191). *Pv*DBP is a main target to use as vaccine candidate since its importance during parasite invasion and its ability to induce antibodies against the parasite’s asexual phases (114, 192).

Serological evaluation in a PNG endemic area has shown that a humoral immune response was common and increased with age, suggesting a possible booster effect regarding antibody response in some cases by repeated exposure to the infection (100). A similar pattern has been observed in an endemic region of Colombia where a positive correlation was found between increased antibody response and patients’ age. Also, an immunologic boost for DBP was found, even in endemic areas having a low transmission level (101). The forgoing shows that DBP_II_ was naturally antigenic in people residing in endemic regions.

Children having high antibody levels against DBP_II_ has been associated with delayed reinfection time with the same *P. vivax* variant; however, such association was not observed when evaluating MSP1–19 (193). In other studies have been observed that naturally acquired neutralizing antibodies against DBP are short-lived, increasing with acute infection, and are strain specific (194, 195).

Antibodies from plasma from naturally exposed people and from animals immunized with recombinant Duffy binding protein (rDBP) have blocked the specific interaction between the *Pv*DBP ligand domain *in vitro* and its receptor on erythrocyte surface; such inhibitory activity has been correlated with antibody titers (196, 197). The forgoing shows DBP’s potential as vaccine candidate due to its essential role as adhesion molecule (196).

The cytokine production of individuals exposed to *P. vivax* was analyzed; IFN-γ, IL-10, and IL-2 induction was observed. The response was seen to depend on individuals’ age and the specific DBP_II_ variant, producing partially acquired immunity to *P. vivax* in these populations (198). Similarly, epitopes mapped from the DBP_II_ critical binding region have produced a humoral response, accompanied by increased antibody levels associated with patients’ increased age, suggesting recognition through repeated infection. Some individuals recognized rDBP_II_ but not linear epitopes, indicating the presence of conformational epitopes; such cases occur regularly in young people or subjects suffering first acute *P. vivax* infection, suggesting that multiple infections are needed for the recognition of linear epitopes (199).

The DBP binding domain (DBP_II_) is polymorphic, tending to compromise the efficacy of any vaccine associated with strain-specific immunity (192). Due to the high rate of polymorphism observed in DBP region II, an in-depth investigation was made of the relative importance of conserved and polymorphic residues in this region by directed mutagenesis. The mutations causing the loss of ligand function were mainly produced in discontinuous groups of conserved residues, while almost all mutations in polymorphic residues did not alter RBC binding (200). Such polymorphism has been seen to have a synergic effect on the antigenic nature of DBP (201). Sera from patients reacted to denatured, non-reduced, and native rDBP, indicating immunogenic conserved linear B-epitopes (100). The immune efficacy of a DBP_II_ vaccine depends on inducing antibodies, and this response should be optimized toward conserved epitopes to protect against *P. vivax* (197).

Since DBP region II epitopes have been shown to be immunogenic, studies have established universal epitopes, which can be presented by different HLA-DR alleles inducing an effective cellular and humoral immune response, making them a candidate for a subunit-based vaccine (202). Antigenicity studies using *Pv*DBP_II_ universal epitopes have shown that lymphoproliferation, IL-6, and IFN-γ production is induced in peripheral blood mononuclear cells (PBMCs) from individuals exposed to infection. Such results have suggested that these epitopes having affinity for HLA-DR molecules can be good components of a vaccine against *P. vivax* (203).

Polymorphisms observed in DARC have also been associated with *P. vivax* infection severity and susceptibility in humans. Individuals with low DARC expression (a single negative allele) have a greater probability of having anti-MSP1 and anti-DBP antibodies than individuals having high DARC expression (double positive alleles). Individuals having high expression of DARC have been found to be associated with greater susceptibility to infection, exhibiting low frequency and magnitude of specific antibody response against *P. vivax* during the blood stage. This could indicate that one of *P. vivax*’s primary mechanisms for evading host immunity works through indirect negative regulation of DARC, influencing the humoral response against erythrocyte invasion and parasite development (204).

### Apical Membrane Antigen-1 (AMA-1)

The AMA-1 in *Plasmodium* is a transmembrane protein, which is localized in the micronemes. It seems to be essential during cell host invasion and is present in all *Plasmodium* species (205, 206). Eight disulfide bonds have been identified in the AMA-1 ectodomain of 66 kDa, defining three different subdomains (DI, DII, and DIII) (207). Immune responses induced by AMA-1 from different *Plasmodium* species have shown potent parasite-inhibitory effects both in animals and *in vitro* thus suggesting AMA-1 as a potential vaccine candidate (208).

*Plasmodium vivax* AMA-1 ectodomain (*PV*66/AMA-1) has been shown to be highly immunogenic in rhesus monkeys, inducing high IgG antibody titers; however, these suffer a rapid decline. A slight reduction in parasitemia has been observed in *P. cynomolgi*-challenged animals previously immunized with *PV*66 (209).

Mice immunized with human adenovirus type 5 and rAMA-1 have produced long-lived specific antibodies (including IgG1 and IgG2a) and memory T-cell proliferative responses. Memory T-cell responses were effector- and central-type, central memory predominating (210). In mice it was observed that response was both Th1 and Th2 following three immunizations and persisted for 1 year following the first immunization. On the other hand, the antibodies produced were capable of recognizing the native protein located on *P. vivax* parasites (211).

When evaluating the immune response against two *Pv*AMA-1 variants (A and B), there were no significant differences regarding the prevalence of IgG response. A marked switching in isotypes, which became increased with age, was also seen. The predominant cytophilic antibodies recognized *Pv*AMA1A (IgG1) and *Pv*AMA1B (IgG1–IgG3). The immune-epidemiological data in this research were similar regarding the two variants, this implied that one of these forms could be used in a universal erythrocyte stage *Pv*AMA-1 antigen-based vaccine (212).

An IgG response was observed in people residing in endemic regions exposed to *P. vivax* in Brazil, IgG1 being the dominant subclass. This antibody response was slightly lower than that observed with MSP1_19_ and increased by 100% in individuals having had more than three episodes. Sequences of *Pv*AMA-1 variable domain from different isolates have been seen to have limited polymorphism in this country (102).

This protein has been seen to be involved in Mrz invasion and contains an extracellular portion containing three different domains (207). When evaluating DI, DII, and DIII, separately or in combination in *P. vivax*-infected individuals, a greater immune response toward proteins containing the domain II was observed. Inhibition assays using the *Pv*AMA-1 ectodomain led to common epitopes being identified within the DI–DII domains, which were recognized by antibodies from people residing in endemic regions. Immunization in mice having the *Pv*AMA-1 ectodomain induced high levels of antibodies, predominantly against DI–II (103).

A linear B-epitope was also identified between amino acids 290–307 (SASDQPTQYEEEMTDYQK) in domain II, this peptide was recognized by sera from individuals naturally infected by *P. vivax* (213).

Recombinant *P. vivax* apical membrane antigen-1 DII was formulated with six adjuvants and was highly immunogenic regardless of the adjuvant used. DII-specific antibodies recognized native AMA-1 protein, demonstrating that it is immunogenic and indicating that this protein region could be evaluated as part of a subunit-based vaccine against malaria caused by *P. vivax* (214).

### Reticulocyte-Binding Proteins (RBP)

Reticulocyte-binding proteins include *Pv*RBP1 and *Pv*RBP2 and their variants *Pv*RBP1a and b and *Pv*RBP2a, b, and c, and other family members (215). RBP1 is a homodimer bound by disulfide bonds, binds non-covalently to RBP2, and forms a protein complex (216). They are colocalized in the apical zone in Mrz micronemes and contain a transmembrane domain toward the C-terminal extreme, possessing repeat regions in *Pv*RBP2 and reticulocyte-binding domains (108, 215, 217–219).

It is thought that RBPs could participate in reticulocyte invasion since no infection by *P. vivax* has been observed in mature erythrocytes (220). Their reticulocyte-binding ability has also been reported, but the specific receptors have yet to be identified (219). It has recently been found that only *Pv*RBP2b binds specifically to reticulocytes (221). Due to their participation in infection, they have been studied as erythrocyte phase vaccine candidates, aimed at blocking Mrz invasion of reticulocytes (218).

High affinity reticulocyte-binding peptides (HARBPs) have been identified, 5 in a fragment from the region I of *Pv*RBP1 NT extreme (222) and 24 throughout the whole protein (223). The highly-conserved region III (between amino acids 1,941–2,229) had the greatest amount of HARBPs (223) and, when used as immunogen, it induced high antibody titers in *Aotus nancymaae* monkeys, able to recognize the full *Pv*RBP1 in parasite lysate. T-lymphocytes became activated following the second and third doses, but no protection was obtained after experimental challenge (224). Due to studies involving other *P. falciparum* proteins showing that highly conserved sequences are not immunogenic, in spite of having high binding capability, it has been suggested that changes must be made in some amino acids to increase the immune response and induce protection based on studies of critical binding residues for HARBPs from region III (224).

Antigenicity studies have found a direct relationship between higher anti-*Pv*RBP1 antibody titers and the number of previous episodes, the time spent residing in an endemic region and age (82, 83, 219, 224). The immune response to *Pv*RBP1 has also been associated with greater IgG1 and IgG3 cytophilic antibody presence against fragments from polymorphic regions (82, 83).

Studies regarding different populations where an acquired response to B-epitopes (107) and various fragments from *Pv*RBP1 and *Pv*RBP2 variants (108, 219) have shown high prevalence of IgG antibodies against fragments from repeat region, the supercoiled helix, and *Pv*RBP1 C-terminal region (107) (Table 2). Regarding the NT region (including the most polymorphic region of the *Pv*RBP1a and b variants) (108), IgG prevalence was intermediate in a population from Thailand, while no antibody response against *Pv*RBP1b was found in a population from the Republic of Korea (219).

On the other hand, *Pv*RBP2 has been seen to have greater antibody prevalence against NT region, repeat region (107), and *Pv*RBP2c variant fragments (219). *Pv*RBP2c is one of the most polymorphic variants, probably having the greatest global distribution, associated with the prevalence of this variant’s recognition (219). *Pv*RBP2b and *Pv*RBP1a have been correlated with lower risk of parasitemia in a cohort study of PNG children (221). Regarding other proteins such as *Pv*DBP, *Pv*RBP antigenicity is much lower (82, 108).

### Antigenicity and/or Immunogenicity of Non-Classical Vaccine Candidates

In spite of the technical limitations involved in studying *P. vivax* proteins, other proteins characterized as being promising vaccine candidates have been studied during the last few years. One such is merozoite surface protein-10 (*Pv*MSP10) having NT and C-terminal regions with two EGF-like domains and a GPI anchor (225). Colombian individuals exposed to *P. vivax* infection have shown reactivity to recombinant *Pv*MSP10; r*Pv*MSP10 has also elicited high antibody titers against the protein in immunized *Aotus* monkeys but no protection after challenge (226). Infected Korean individuals had 42% IgG prevalence, IgG1 and IgG3 antibodies predominating. Immunized mice have shown a Th1 response-biased immune response (227).

The *Pv*34 protein was characterized based on homology of the *P. falciparum Pf*34 protein, and antigenicity was evaluated in PBMCs from individuals previously exposed to infection. Stimulation with r*Pv*34 induced proliferation in 71% of individuals and high IL-2, IFN-γ, and IL-4 production (Th1/Th2 profile), such response being attributed to recognition of T- and B-epitopes responsible for a combined immune response (228).

Another protein characterized was *Pv*RON-1, based on its homologous *Pf*ASP protein in *P. falciparum*. This protein’s antigenicity was evaluated using sera from people having had previous *P. vivax* infection. The results showed that *Pv*RON-1 was expressed during natural infection and could generate an antibody response in the host (229).

An antigenicity and immunogenicity study of *P. vivax* rhoptry-associated leucine (Leu) zipper-like protein-1 (*Pv*RALP-1) was carried out on patient serum samples and immunized mice. *Pv*RALP-1 was recognized in samples from patient sera, IgG1 and IgG3 being the predominant subclasses, although without significant differences with the other subclasses; Th1/Th2 response was balanced in immunized mice (230).

The CelTOS microneme protein characterized and tested for *P. falciparum*, having 98% homology with *P. vivax*, should be considered for further studies since cross-species protection has been demonstrated in preclinical studies (231). It has been described as vaccine target by blocking transmission infection or preerythrocytic stage due to its cell traversal function in Spz and ookinetes (232). A recent study proved ~20% prevalence of antibodies against *Pv*CelTOS in a Thai population (233). More studies related to immunogenicity, and antigenicity potential are needed and should involve new proteins characterized during the last few years, related to host cell invasion during different life cycle stages, such as rhoptry neck proteins.

## Transmission Blocking *P. vivax* Vaccine Candidates

One of the methodologies used for controlling malaria infection has been the search for transmission blocking vaccines, through strategies for preventing ookinete development in the vector (234). The assays conducted for transmission blocking have been focused on two main proteins, *Pv*s25 and *Pv*s28, expressed on gametocyte surface. Anti-sera have recognized *Pv*25 in zygotes and mature ookinetes, and *Pv*28 more in mature ookinetes (235).

Immunogenicity tested with recombinant proteins *Pv*s25 and *Pv*s28 in mice has shown a splenic T-cell proliferative response. Anti-sera from mice immunized with a *Pv*s25–28 chimera had a high antibody titer compared to mice immunized with *Pv*s25 or *Pv*s28 alone. Anti-*Pv*s25–28 and anti-*Pv*s25 had higher transmission blocking than anti-*Pv*s28 (235, 236).

Studies had demonstrated that *P. vivax Sal*I strain recombinant *Pv*s25 and *Pv*s28 had transmission blocking capability, even with natural isolates, thus overcoming genetic polymorphism between isolates (237). Higher transmission blocking has been described as a direct function of antibody titers in sera (238–240).

Different vaccination schemes have been tested, varying adjuvant, dose, expression system (148, 237, 238, 240, 241). Phase I clinical trials determining security and immunogenicity in humans have shown high transmission blocking capability in humans, demonstrating these antigens’ potential as vaccine candidates (147, 148).

## Conclusion

This review has summarized immune responses induced by *P. vivax* vaccine candidates, which are essential in host cell invasion. Classical vaccine development has been focused on immunodominant antigens such as sporozoite and MSPs, which are recognized by sera from partially protected individuals who are naturally exposed to infection. However, surface proteins, for example *Pv*MSP1 and *Pv*CSP, have high allelic polymorphism (164, 242, 243) and are under positive selection by the immune response. After several natural infections, many of these epitopes have shown an ability to generate a strong immune response in individuals without clinical symptoms, showing an association with IFN-γ effector T-cell activation and generation of cytophilic antibody subclasses. Similar immune responses have been observed in animal models immunized with these polymorphic immunodominant antigens. Nevertheless, the success for this kind of vaccines has been limited, since the cross protectivity obtained for the remaining strains is very low and induces a short-lived immune response (244, 245). Moreover, parasites change their cell targets and molecules during preerythrocytic, erythrocytic, and sexual stages, and single-antigen/single-stage vaccines do not induce sterile protection. It has been observed that *P. falciparum* parasites hide their amino acid conserved domains of the proteins involved in the invasion of host cells, showing immune dominant and polymorphic epitopes to the immune system (246).

Other methodologies are needed to solve these kinds of issue. An alternative is to develop an antimalarial vaccine (246), focused on synthetic peptides designed on conserved regions of Spz and Mrz proteins having high hepatic cell or RBC-binding ability. Although these peptides are not immunogenic, *P. falciparum* and *P. vivax* studies have shown that such peptides can be modified by changing their critical RBC-binding residues for others having similar mass but opposite polarity, making them highly immunogenic and protective (247, 248).

Another problem in the development of an antimalarial vaccine concerns the many haplotypes present in the exposed population. The HABPs can also be modified that fit properly inside the peptide-binding region of MHCII. In studies with *P. falciparum* with a MSP-2 HABP that has been modified to bind to HLA-DRβ1*0403 molecules with high affinity, it was shown that *Aotus* monkeys bearing HLA-DRβ1*0403-like molecules, produced high antibody titers with sterile immunity after challenge with *P. falciparum* FVO. This was a proof-of-concept immune protection-inducing protein structures demonstrating that specifically modified HABPs are able to induce sterile protection against malaria by engaging the proper TCR/pMHCII interactions (249).

Evaluation of conserved epitopes and non-immunodominant antigens important in parasite adhesion and invasion of erythrocytes should be prioritized for multistage, multi-epitope, minimal subunit-based, chemically synthesized antimalarial development, covering a large part of the HLA-DRβ1* population in endemic areas to protect them against malarial parasites.

## Author Contributions

CL conceived the work and drafted the manuscript; YY-P drafted the manuscript and designed the figures; NH-E and DD-A drafted the manuscript; MP critically revised the manuscript for important intellectual content. All authors have revised the manuscript and given their approval for the version to be submitted.

## Conflict of Interest Statement

The authors declare that the research was conducted in the absence of any commercial or financial relationships that could be construed as a potential conflict of interest.

## Abbreviations

Spz, sporozoites; RBC, red blood cells; NT, N-terminal; pexel/VTS, *Plasmodium* export element/vacuolar translocation signal; Mrz, merozoites; NFkβ, nuclear factor kappa B; PRRs, pattern recognition receptors; PAMPs, pathogen-associated molecular patterns; MHC, major histocompatibility complex; NK, natural killer; IFN-γ, interferon gamma; TNF, tumor necrosis factor; LDH, lactate dehydrogenase; TAT, thrombin–antithrombin III; HNE, human neutrophil elastase; IL, interleukin; Th1, T helper 1; Th2, T helper 2; CXCL9, chemokine (C–X–C motif) ligand 9; CCL2, chemokine (C–C motif) ligand 2; CCL5, chemokine (C–C motif) ligand 5; Ig, immunoglobulin; i.v., intravenously; HLA, human leukocyte antigen; CSP, circumsporozoite protein; TRAP, thrombospondin-related adhesive protein; GPI, glycophosphatidylinositol; PNG, Papua New Guinea; LSP, long synthetic peptides; TLR, toll-like receptor; CpG, cytosine and guanine separated by one phosphate; GLA, glucopyranosyl lipid A; TSR, thrombospondin type 1 repeat; MSP, merozoite surface protein; ELISA, enzyme-linked immunosorbent assay; EGF, epidermal growth factor; HABPs, high activity binding peptides; AMA-1, apical membrane antigen-1; r*Pv*AMA-1, recombinant *Plasmodium vivax* apical membrane antigen-1; FcγR-Fc, Fc-gamma receptors; PP, poly-proline; TCR, T-lymphocyte receptor; ADCI, antibody-dependent cell-mediated inhibition; DBP, Duffy binding protein; DARC, Duffy antigen receptor for chemokines; rDBP, recombinant Duffy binding protein; PBMC, peripheral blood mononuclear cell; RBP, reticulocyte-binding proteins; HARBPs, high affinity reticulocyte-binding peptides.
